# Digital Personal Health Coaching Platform for Promoting Human Papillomavirus Infection Vaccinations and Cancer Prevention: Knowledge Graph-Based Recommendation System

**DOI:** 10.2196/50210

**Published:** 2023-11-15

**Authors:** Nariman Ammar, Olufunto A Olusanya, Chad Melton, Lokesh Chinthala, Xiaolei Huang, Brianna M White, Arash Shaban-Nejad

**Affiliations:** 1 Center for Biomedical Informatics, Department of Pediatrics College of Medicine University of Tennessee Health Science Center Memphis, TN United States; 2 School of Information Technology Illinois State University Normal, IL United States; 3 Ochsner Xavier Institute for Health Equity and Research Ochsner Clinic Foundation New Orleans, LA United States; 4 Bredesen Center for Interdisciplinary Research and Graduate Education University of Tennessee Knoxville, TN United States; 5 Department of Computer Science University of Memphis Memphis, TN United States

**Keywords:** health information exchanges, knowledge graphs, recommender systems, personal health libraries, vaccine promotion, cancer prevention, personal health informatics

## Abstract

**Background:**

Health promotion can empower populations to gain more control over their well-being by using digital interventions that focus on preventing the root causes of diseases. Digital platforms for personalized health coaching can improve health literacy and information-seeking behavior, leading to better health outcomes. Personal health records have been designed to enhance patients’ self-management of a disease or condition. Existing personal health records have been mostly designed and deployed as a supplementary service that acts as views into electronic health records.

**Objective:**

We aim to overcome some of the limitations of electronic health records. This study aims to design and develop a personal health library (PHL) that generates personalized recommendations for human papillomavirus (HPV) vaccine promotion and cancer prevention.

**Methods:**

We have designed a proof-of-concept prototype of the Digital Personal Health Librarian, which leverages machine learning; natural language processing; and several innovative technological infrastructures, including the Semantic Web, social linked data, web application programming interfaces, and hypermedia-based discovery, to generate a personal health knowledge graph.

**Results:**

We have designed and implemented a proof-of-the-concept prototype to showcase and demonstrate how the PHL can be used to store an individual’s health data, for example, a personal health knowledge graph. This is integrated with web-scale knowledge to support HPV vaccine promotion and prevent HPV-associated cancers among adolescents and their caregivers. We also demonstrated how the Digital Personal Health Librarian uses the PHL to provide evidence-based insights and knowledge-driven explanations that are personalized and inform health decision-making.

**Conclusions:**

Digital platforms such as the PHL can be instrumental in improving precision health promotion and education strategies that address population-specific needs (ie, health literacy, digital competency, and language barriers) and empower individuals by facilitating knowledge acquisition to make healthy choices.

## Introduction

### Background

Precision health promotion empowers populations and communities to choose health-related behaviors that allow them to gain more control over their health and well-being through a variety of socio-behavioral, environmental, and economic interventions [1]. The World Health Organization conceptualizes health promotion as “the process of enabling people to increase control over and to improve their health” [2]. Rather than emphasizing treatments and palliation, health promotion ultimately reduces the risks of chronic diseases by targeting and preventing the root causes of these diseases [1]. Some of the main elements of health promotion [1] are to (1) improve population health literacy and knowledge acquisition to ensure healthy behavioral choices; (2) create healthy cities through healthy urban planning to mitigate; and (3) prioritize good policies and better health decision-making that facilitates, for example, vaccine uptake [3]. Vaccinations have deterred countless incidences of vaccine-preventable illnesses and some infectious diseases across the globe.

Consequently, vaccines are proven to be the most effective and cost-beneficial public health intervention for improving health outcomes and saving lives [4]. While lifestyle factors, including alcohol consumption, cigarette smoking, and prolonged sun exposure, are recognized for increasing the risk of cancer, vaccination against infectious agents such as viruses and bacteria does not receive the same level of attention. For instance, approximately 90% of human papillomavirus (HPV)–associated cancers found in the cervix, vagina, vulva, penis, anus, rectum, and oropharynx are preventable through the uptake of the HPV vaccine [5].

Despite the HPV vaccine’s effectiveness at preventing cancer-causing infections and precancers, more than 45,000 incident cases of HPV-associated cancer diagnoses are reported in the United States annually [6,7]. Although secondary cancer prevention methods (ie, Papanicolaou tests) can detect some types of HPV-related cancers, other types associated with infection are left undetected due to the absence of screening measures (ie, head and neck cancers) [8]. As a result, the Centers for Disease Control and Prevention (CDC) Advisory Committee on Immunization Practice recommends a 2-dose vaccine series for male and female adolescents aged 9 to 12 years in the United States [9]. Since vaccinations are only a prophylactic measure and cannot treat HPV infections, early vaccination before infection exposure is key to cancer prevention [9]. Though most effective if administered in early childhood, all individuals through the age of 26 years who have not been fully vaccinated should obtain the HPV vaccine [10].

More recently, vaccination uptake behavior has been adversely impacted by growing parental vaccine hesitancy due to religious and philosophical beliefs that oppose vaccinations, infodemic, spikes in vaccine misinformation, vitriolic and politicized debates about COVID-19 vaccines, COVID-19 pandemic-related disruptions to routine childhood vaccination services, socio-contextual and environmental barriers, public mistrust in vaccine safety and efficacy, perceptions about vaccine side effects, as well as the lack of or inadequate health insurance and health provider recommendations [11,12].

Accordingly, it is pertinent that novel techniques that support precision health promotion are implemented to increase vaccination uptake behaviors. The application of digital educational interventions for personalized health coaching can generate customized recommendations in a case-by-case manner, improve health literacy and knowledge acquisition, optimize information-seeking behavior, promote healthy behavior (eg, HPV vaccine uptake; sexually transmitted infection [STI], HPV, or genital warts testing; and cervical cancer screening), and lead to better population health outcomes (eg, HPV-associated cancer prevention) [13,14]. Advances in artificial intelligence (AI) and digital technologies have revolutionized several applications in health care [15], promoting and enhancing personalized patient care. For instance, formal semantic data and knowledge representation techniques have enabled the implementation of explainable AI systems by providing extra insights for learning and explanations based on hybrid approaches to AI that combine probabilistic machine learning with symbolic, rule-based reasoning. Those approaches use content and contextual knowledge encoded in ontologies to build and enrich personal health knowledge graphs (PHKGs).

### Role of Digital Technologies in Improving Health and the Delivery of Care

Digital health technologies such as mobile health (mHealth) and mobile medical applications, health information technology, smart devices, wearable sensors, wireless medical devices, and telemedicine or telehealth have revolutionized health care systems [15]. With the use and application of AI and machine learning, these technologies provide scientists, health care providers, and public health officials with the infrastructure to gather, manage, and interpret heterogeneous complex data sets to provide real-time recommendations for health decision-making and response [16]. In addition to rapid analysis, digital health solutions could enable personalized and patient-centered approaches to health care management, giving providers better insight into interpreting biological and social markers that could accurately predict actual health status [17]. The application of these innovative tools could optimize decision-making with the ability to tailor treatment and therapies to patient-specific characteristics such as disease history, genetic profile, psychosocial attributes, diagnostic imaging information, or prior treatment responses [18].

### Design of Digital Health Solutions to Promote Patient Engagement

The continual expansion of digital health solutions provides the opportunity for patient empowerment and the ability to participate fully in their health care decision-making processes. Patient engagement has historically been limited to patient-provider interactions, with little room for self-management of both chronic and acute conditions [18]. Notably, findings from numerous studies demonstrate that patient empowerment and engagement are key factors in achieving positive health outcomes [19-22]. With the development of digital technologies such as wearable smart devices and sensors, individuals can track essential vitals such as heart rate and blood pressure and even identify cardiac arrhythmias by generating single-lead electrocardiograms at the touch of a finger [23]. However, despite a well-documented public desire for technological advancements in health management [24,25], digital health solutions are often not effectively used [19]. This underuse is attributed to numerous challenges of current digital health solutions, such as digital health disparities (ie, the lack of access to digital health services), unsustainable costs, insufficient digital skills, low level of end user satisfaction, poor user experience, digital navigation difficulties, privacy concerns, varying levels of digital and health literacy, and unreliable provider recommendations for use [26]. To successfully empower patients to take a stake in their health, effective digital health solutions should be designed with end users in mind to fully engage and educate patients on disease management and monitoring, digital health literacy, health information-seeking behaviors, and vaccine safety or efficacy.

## Methods

### Study Design

We have recently proposed a paradigm-shifting design for building a personal health library (PHL) [27,28] and demonstrated how the PHL can serve as a knowledge infrastructure for building mHealth apps for the self-management of diabetes [29]. We have also implemented a hybrid evidence-based and knowledge-driven explainable AI recommender and digital assistant for mental health surveillance [30]. The PHL integrates health data with multidimensional and multimodal nonhealth data, including observations of daily living (ODL) and population-level social determinants of health (SDoH). Therefore, it enables both patients and health care professionals to make evidence-based decisions by integrating 3 components: clinical expertise, research literature, and patient preferences [31]. The approach used to build the PHL leverages several innovative technological infrastructures, including Semantic Web, the web application programming interface (API), hypermedia-based discovery, and the social linked data [32] platform to build a privacy-aware, decentralized, yet linked architecture that enables seamless communication among health professionals across different organizations and platforms.

**Figure 1:**
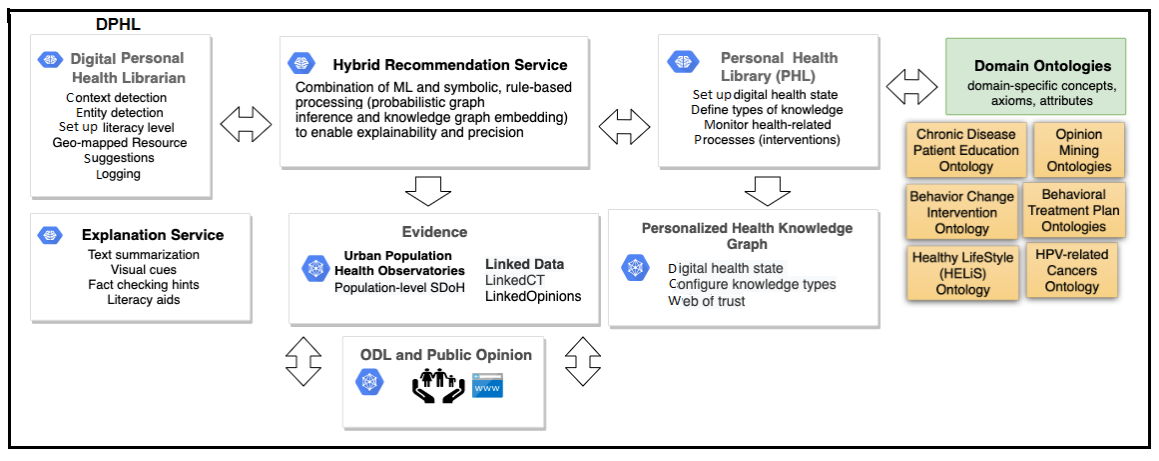
A DPHL that uses the types of knowledge stored in the PHL to provide hybrid recommendations and explanations. DPHL: Digital Personal Health Librarian; HPV: human papillomavirus; ML: machine learning; ODL: observations of daily living; PHL: personal health library; SDoH: social determinants of health.

In this paper, we demonstrate how the Digital Personal Health Librarian (DPHL; Figure 1) can use the PHL to generate personalized recommendations, enhance health literacy and knowledge acquisition, optimize information-seeking behavior, promote healthy behavior (eg, HPV vaccine uptake; STI, HPV, or genital warts testing; safe sexual practices; and cervical cancer screening) and overcome false perceptions and misinformation regarding HPV infection and vaccine hesitancy among adolescents and their caregivers. The PHL is currently in its prototype stage and is actively being developed to optimize its implementation and functionalities.

### Health Coaching Scenario

**Figure 2:**
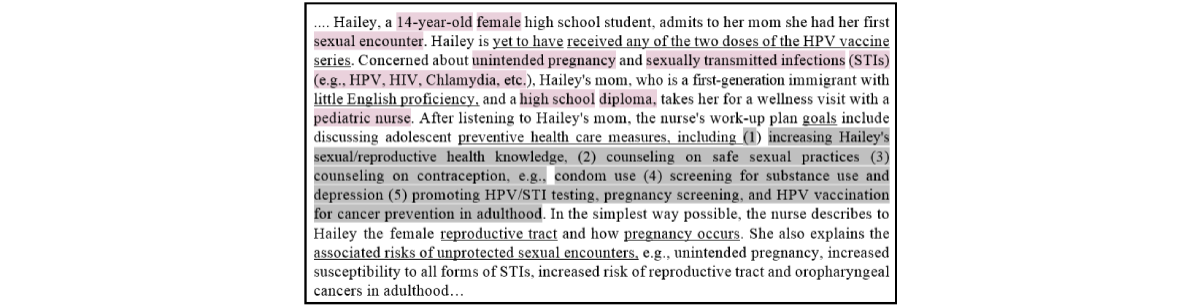
A health coaching scenario. HPV: human papillomavirus.

In Figure 2, we showcase a scenario from the vaccine promotion, education, and cancer prevention domain. Our goal is to identify issues and needs that can be resolved with the help of a digital intervention. This intervention could improve knowledge acquisition, promote safe sexual practices, increase HPV vaccine uptake, and facilitate the adoption of routine HPV-based screening and testing protocols.

The scenario shows that in a real-world setting, several types of knowledge can be captured through a conversation with a health coach or a clinician. For example, engaging in unprotected sex is considered risky behavior that increases the likelihood of unintended pregnancies, HPV infections, HIV, and other STIs. In addition, other types of knowledge are brought up during the conversation. For example, the fact that Hailey’s mom cannot speak English fluently and Hailey’s unvaccinated status serve as upstream social determinants of risk factors. We wish to simulate this conversation in a digital health intervention platform, which we will explain next.

### Hybrid DPHL

Figure 1 shows the main components of our DPHL, which simulates a digital health coach. The app passes detected entity types and contextual parameter values to backend services, including the recommendation service, the explanation service, and the PHKG generation component. Based on our scenario, the PHL needs to capture knowledge from relevant domains of interest by reusing several domain ontologies and controlled vocabularies focusing on patients’ education (eg, Chronic Disease Patient Education Ontology [33]), behavior change (eg, Behavior Change Ontology [34]), and supporting healthy lifestyles (eg, HELiS Ontology [35]). It also uses other ontologies for knowledge misconceptions. Further, if Hailey wants to receive warnings about misconceptions discussed over social media platforms, the PHL can collect such knowledge by using concepts from opinion-mining ontologies (eg, Marl standardized data schema or ontology [36]). By accessing dynamic knowledge discovered through the PHL, the DPHL can provide real-time hybrid recommendations that are both content and context based. For example, based on our scenario, the DPHLP can provide recommendations on HPV-associated cancer preventive measures, including safe sexual practices, HPV vaccine uptake, and routine HPV testing.

### Setting Up the PHL

We explain the steps by which the PHL processes Hailey’s digital health state, including the following: (1) setting up her digital health profile and specifying her web of trusted agents, (2) specifying types of knowledge (concepts) she would like to maintain in the library, (3) populating concepts with content based on evidence, and (4) using context to personalize the collected knowledge-seeking for resources considering SDoH and ODL.

### Setting Up the Web of Trust

It is apparent from the scenario that Hailey may need to rely on (and trust) several individuals, including her mom, the health coach, and the clinician. A personal health coach is qualified to discuss adolescent preventive health care measures, for example, (1) increase Hailey’s sexual or reproductive health knowledge; (2) promote HPV vaccine uptake and HPV or STI testing as cancer preventive measures; (3) counsel on safe sexual practices; (4) counsel on contraception, for example, condom use; and (5) screen substance use or depression. The coach can also counsel on the adverse outcomes of HPV or STI and unprotected sex. On the other hand, only the clinician can administer the vaccines and obtain samples for STI tests. Next, we explain how the above scenario reflects needs related to the 4 HPV-associated cancer preventive measures: safe sexual practices, HPV vaccine uptake, routine HPV testing, and cancer screenings.

### Generating the PHKG

**Figure 3:**
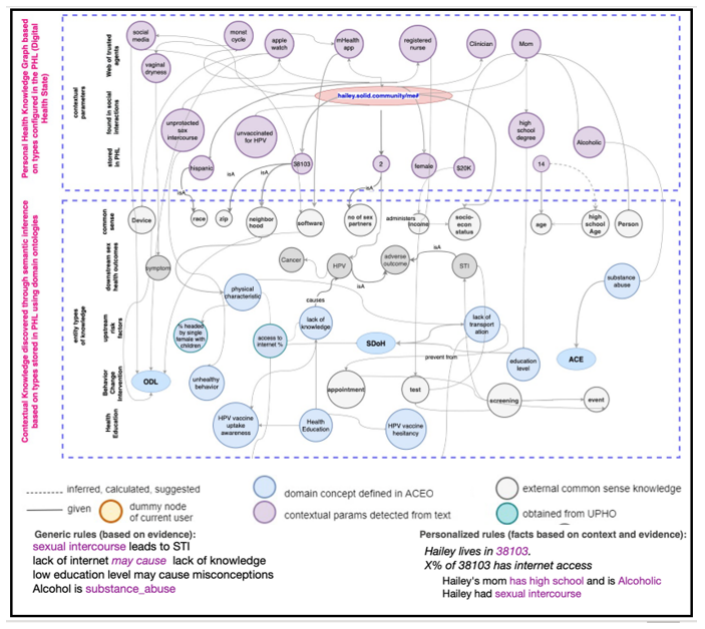
A PHKG that represents contextual parameters detected in conversations, their corresponding types of knowledge stored in the PHL, as well as new types and causal relations inferred using concept hierarchies and axioms encoded in ontologies. ACE: adverse childhood experience; ACEO/PopHR: ACEs Ontology/population health record; HPV: human papillomavirus infection; mHealth: mobile health; ODL: observations of daily living; PHKG: personal health knowledge graph; PHL: personal health library; SDoH: social determinants of health; STI: sexually transmitted infection; UPHO: Urban Population Health Observatory.

The PHL then uses those types of knowledge to construct and enrich a PHKG for Hailey (Figure 3). The knowledge graph (KG) refinement is performed in three main iterations: (1) first, the PHL uses the information provided by Hailey as contextual entry points to the KG and links them to entity types already stored in the library; (2) it then infers extra knowledge based on concept hierarchies and object properties stored in different domain ontologies; and (3) then, it populates the inferred concepts with evidence from local (city-level urban observatories) or public knowledge (opinions and research).

### Populating PHKG With Evidence

**Figure 4:**
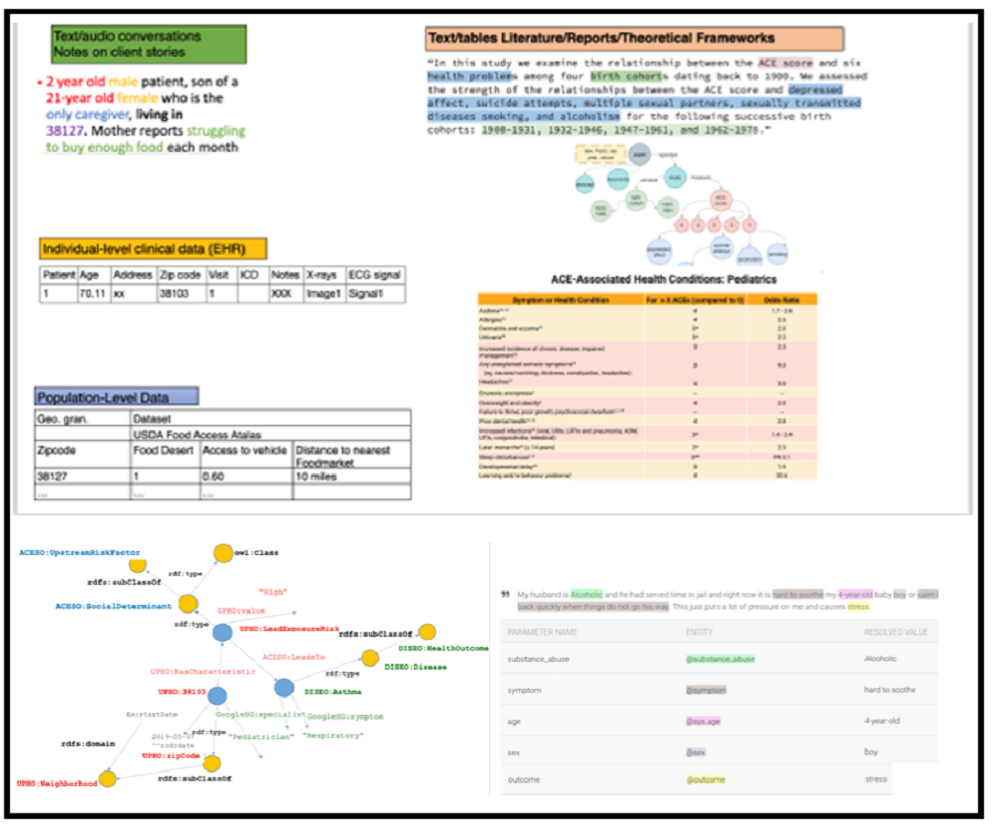
(1) Multimodal multidimensional data integration from multiple sources, including text detected in conversations, text, or tabular data from literature or reports, individual-level and population-level data, as well as data stored in graphs (focusing on adverse childhood experiences screening scenarios as an example). (2) NLP is applied to text to detect domain concepts based on domain ontologies to generate RDF graphs. ACE: adverse childhood experience; EHR: electronic health record; NLP: natural language processing; RDF: resource description framework.

The PHL captures context and collects evidence from several sources of data and knowledge (Figure 4).

- Individual-level patient preferences and ODL: data from physical activity trackers and electronic subscription behaviors are collected.
- Individual-level data from electronic health records: a huge amount of data in electronic health record (EHR)–specific standards are made available for multiple purposes. In addition to using EHR data for clinical practice, EHR data warehousing can provide longitudinal data on the patient care received over time and benefit hypothesis testing, population screening, clinical trial feasibility evaluation, and analyzing treatment pathways. Furthermore, the vaccination and immunization history data can also be leveraged from structured and unstructured EHR data. The EHR data can also be combined with immunization information systems to track the immunizations received in multiple health care facilities. We plan to deploy text-mining techniques to extract physiological and immunization-related information from these unstructured data.
- Multidimensional population-level SDoH: the PHL aligns the individual-level data with population-level SDoH data regarding neighborhood characteristics from public sources (eg, from the US Census Bureau and local partners).
- Trusted sources of evidence: to collect evidence and enrich the knowledge base, the PHL connects the data and concepts with available literature (eg, PubMed KG [37] and LinkedCT [38]). It also collects data from the CDC, which maintains a data repository of concepts related to pregnancy and vaccination, sexually transmitted diseases, smoking and tobacco use, teen vaccinations, vaccinations, and web metrics and makes them accessible through the Socrata Open Data API. Using this API, we aim to use the data sets on teen vaccinations.
- Public opinions: online social media has been proven to be a valuable resource for vaccine attitude surveillance [39]. We intend to collect vaccine-related data from the Reddit information-sharing social media platform, which is composed of user-created communities (subreddits) in which members adhere to a set of regulations. Subreddit members have the option to post links, images, videos, and text. Anonymous community members then typically upvote or downvote a post based on their opinion of the quality of that post. Depending on the distribution of votes, posts are classified as hot, new, rising, and controversial. The most popular posts within each category are then promoted. Users can also post comments, which undergo the same vote ranking mechanism. The upvote or downvote system within Reddit is intended to increase the quality of the posts to minimize nonrelevant material. We will collect and harvest textual data through the Reddit API in conjunction with the Python Reddit API Wrapper. We will then conduct sentiment analysis and content analysis and apply semantic techniques to develop an optimal system to identify misinformation within these communities.

The PHL integrates global knowledge from the above sources with local knowledge in Hailey’s library. Some of the collected knowledge is already in semantic representation (eg, LinkedCT [38], a ClinicalTrials.gov linked data set that defines concepts related to diseases and interventions). For unstructured data, the PHL transforms the collected evidence into a machine-readable format using Semantic Web technologies and links them to semantic conceptual hierarchies of knowledge types stored in the library.

### Ethical Considerations

This study does not include any real patient data, identifiable personal information, or any human material, and therefore, it is not human subjects research and did not require ethics committee approval. All information presented in case scenarios are solely generated for the sake of clarity in describing and demonstrating functionalities provided by the PHL.

## Results

**Figure 5:**
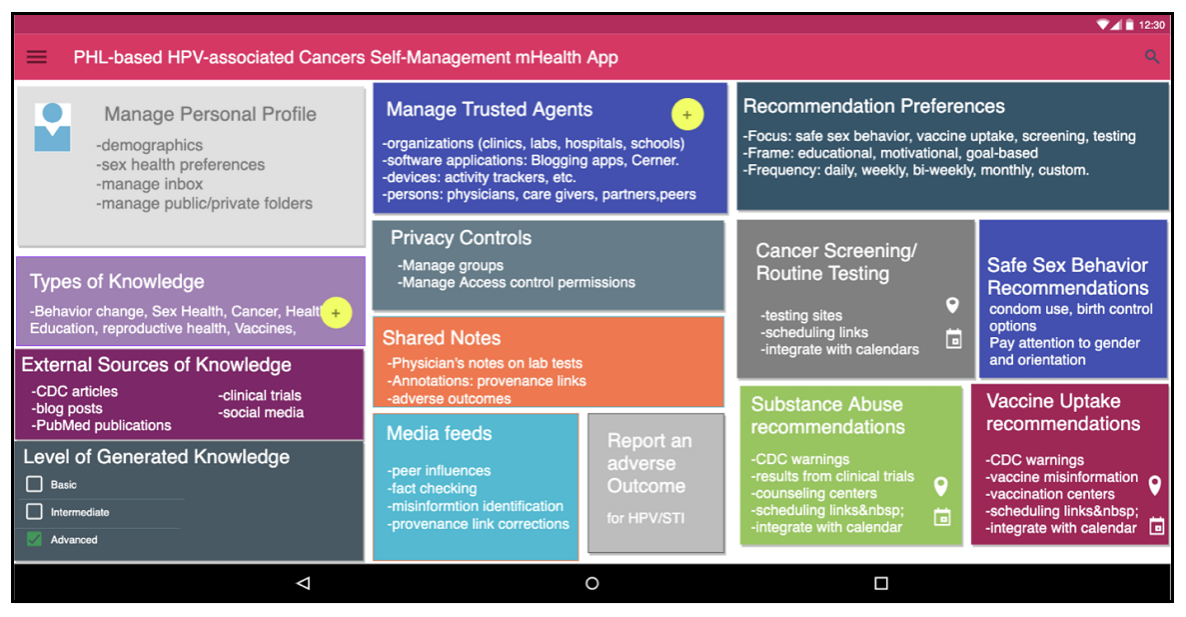
Capabilities provided by the DPHL mHealth app include preferences to set up their profile, indicate types of knowledge they are interested in, their level of experience, and their trusted agents as well as privacy controls. The app will generate recommendations based on their preferences and they can choose different goals. They also obtain reminders of cancer screening and routine testing as well as vaccines. CDC: Centers for Disease Control and Prevention; DPHL: Digital Personal Health Librarian; HPV: human papillomavirus; mHealth: mobile health; PHL: personal health library; STI: sexually transmitted infection.

The preliminary prototype design and capabilities provided by the DPHL mHealth app are shown in Figure 5. Some of the features include preferences to set up their profile, indicating the types of knowledge they are interested in, their level of experience, and their trusted agents, as well as privacy controls. The app will generate recommendations based on their preferences and they can choose different goals. They also obtain reminders of cancer screening and routine testing as well as vaccines.

## Discussion

Health promotion empowers individuals by facilitating the acquisition of knowledge and information to make healthy choices and decisions. In this work, we have proposed a paradigm-shifting design of the PHL that improves the maturity of EHR implementations. The proposed PHL could integrate and leverage complex individual- and population-level data from several multidimensional sources such as EHR systems (health histories, prescriptions, laboratory results, and demographic information); consumer health applications, wearable sensors, and devices; US Census Bureau (eg, SDoH indicators and neighborhood characteristics); PubMed KG and LinkedCT; CDC data repository; and social media platforms, for example, Reddit, to offer patients a personalized health assessment and wellness plan.

While the primary focus of this paper is on promoting HPV vaccinations and cancer screening with the PHL, this app could also address barriers to the receipt of other vaccinations, including tetanus, diphtheria, and acellular pertussis; viral hepatitis (hepatitis A, hepatitis B, and hepatitis C); meningococcal; influenza; and COVID-19. Additionally, the proposed PHL could address obstacles to health care delivery services, including limited access to care, poor knowledge and the lack of information, and the lack of provider’s recommendation, to improve vaccination outcomes.

The digital PHL could provide personalized recommendations in the form of either follow-up interventions with associated geo-mapped resource suggestions (eg, clinics near patient or consumer zip code) or follow-up questions to gather further knowledge. It could also provide digital content in the PHL in the form of summarized textual educational material, visual aids, and explanations of recommendations (by providing source links to evidence). Further, similar to grammar checkers, semantic fact-checking can enable patients or consumers to identify and correct misconceptions using fact-checking links. The PHL can also adjust personalized recommendations to the end user’s level of literacy.

The PHL uses advances in Semantic Web and AI technologies to augment EHR implementation capabilities and fill existing gaps in EHR knowledge acquisition and explanation. We have demonstrated how the DPHL can use the PHL for personalized self-management, including education and precision health promotion. Our proposed PHL could empower patients and caregivers by giving them a central role in their own decision-making process. Moreover, it could equip health providers with informatics tools to proactively collect and effectively interpret patient data in a contextualized and personalized manner. By offering the PHL functionality as an open service, our proposed platform can encourage the development of third-party applications and services.

The PHL has the potential to offer motivational technological support for projects that span different domains of interest beyond the HPV case study discussed in this paper. We expect that this technology will complement conventional clinical care and management for patients with a variety of other comorbidities and chronic conditions by providing tailored, personalized messaging to improve health-related behaviors and enhance treatment plans. The PHL is currently at the prototype stage and is under active development to implement and optimize its full functionalities. A formal user experience assessment using qualitative focus group interviews and quantitative surveys has been planned to be conducted after the system’s full implementation and performance. The assessment of end user experience could be used to gain insights into application effectiveness, functionality, usability, and popularity.

## Abbreviations

AI: artificial intelligence
API: application programming interface
CDC: Centers for Disease Control and Prevention
DPHL: Digital Personal Health Librarian
EHR: electronic health record
HPV: human papillomavirus
KG: knowledge graph
mHealth: mobile health
ODL: observations of daily living
PHKG: personal health knowledge graph
PHL: personal health library
SDoH: social determinants of health
STI: sexually transmitted infection

## Data Availability

Data sharing does not apply to this paper as no data sets were generated or analyzed during this study.

## References

[1] Shaban-Nejad A, Michalowski M, Peek N, Brownstein JS, Buckeridge DL (2020). Seven pillars of precision digital health and medicine. Artif Intell Med.

[2] World Health Organization, Canadian Public Health Association (1987). Ottawa charter for health promotion. Bull Pan Am Health Organ.

[3] (2016). Health promotion. World Health Organization.

[4] Kazi AM (2017). The role of mobile phone-based interventions to improve routine childhood immunisation coverage. Lancet Glob Health.

[5] Printz C (2015). FDA approves Gardasil 9 for more types of HPV. Cancer.

[6] (2021). How many cancers are linked with HPV each year?. Centers for Disease Control and Prevention.

[7] Liao CI, Francoeur AA, Kapp DS, Caesar MAP, Huh WK, Chan JK (2022). Trends in human papillomavirus-associated cancers, demographic characteristics, and vaccinations in the US, 2001-2017. JAMA Netw Open.

[8] Shapiro GK (2022). HPV vaccination: an underused strategy for the prevention of cancer. Curr Oncol.

[9] Glatman-Freedman A, Nichols K (2012). The effect of social determinants on immunization programs. Hum Vaccin Immunother.

[10] (2021). HPV vaccine. Centers for Disease Control and Prevention.

[11] Meites E, Szilagyi PG, Chesson HW, Unger ER, Romero JR, Markowitz LE (2019). Human papillomavirus vaccination for adults: updated recommendations of the advisory committee on immunization practices. Centers for Disease Control and Prevention.

[12] Olusanya OA, Bednarczyk RA, Davis RL, Shaban-Nejad A (2021). Addressing parental vaccine hesitancy and other barriers to childhood/adolescent vaccination uptake during the coronavirus (COVID-19) pandemic. Front Immunol.

[13] Michalowski M, Austin RR, Mathiason MA, Maganti S, Schorr E, Monsen KA (2018). Relationships among interventions and health literacy outcomes for sub-populations: a data-driven approach. Kontakt.

[14] Olusanya OA, Ammar N, Davis RL, Bednarczyk RA, Shaban-Nejad A (2021). A digital personal health library for enabling precision health promotion to prevent human papilloma virus-associated cancers. Front Digit Health.

[15] Shaban-Nejad A, Michalowski M, Buckeridge DL (2018). Health intelligence: how artificial intelligence transforms population and personalized health. NPJ Digit Med.

[16] Ahmed Z, Mohamed K, Zeeshan S, Dong X (2020). Artificial intelligence with multi-functional machine learning platform development for better healthcare and precision medicine. Database (Oxford).

[17] Bohr A, Memarzadeh K (2020). The rise of artificial intelligence in healthcare applications. Artificial Intelligence in Healthcare.

[18] Di Minno G, Tremoli E (2017). Tailoring of medical treatment: hemostasis and thrombosis towards precision medicine. Haematologica.

[19] Birnbaum F, Lewis D, Rosen RK, Ranney ML (2015). Patient engagement and the design of digital health. Acad Emerg Med.

[20] Schmidt M, Eckardt R, Scholtz K, Neuner B, von Dossow-Hanfstingl V, Sehouli J, Stief CG, Wernecke KD, Spies CD (2015). Patient empowerment improved perioperative quality of care in cancer patients aged ≥ 65 years—a randomized controlled trial. PLoS One.

[21] Hibbard JH, Greene J (2013). What the evidence shows about patient activation: better health outcomes and care experiences; fewer data on costs. Health Aff (Millwood).

[22] Chatzimarkakis J (2010). Why patients should be more empowered: a European perspective on lessons learned in the management of diabetes. J Diabetes Sci Technol.

[23] Spaccarotella CAM, Polimeni A, Migliarino S, Principe E, Curcio A, Mongiardo A, Sorrentino S, de Rosa S, Indolfi C (2020). Multichannel electrocardiograms obtained by a smartwatch for the diagnosis of ST-segment changes. JAMA Cardiol.

[24] Ranney ML, Choo EK, Wang Y, Baum A, Clark MA, Mello MJ (2012). Emergency department patients' preferences for technology-based behavioral interventions. Ann Emerg Med.

[25] Brittne KN (2020). Older adults keep pace on tech usage: 2020 tech trends of the 50+. AARP Research.

[26] Affinito L, Fontanella A, Montano N, Brucato A (2022). How physicians can empower patients with digital tools. J Public Health (Berl.).

[27] Ammar N, Bailey JE, Davis RL, Shaban-Nejad A, Shaban-Nejad A, Buckeridge DL, Michalowski M (2020). Implementation of a personal health library (PHL) to support chronic diseases self-management. Explainable AI in Healthcare and Medicine: Building a Culture of Transparency and Accountability.

[28] Ammar N, Bailey JE, Davis RL, Shaban-Nejad A (2020). The personal health library: a single point of secure access to patient digital health information. Stud Health Technol Inform.

[29] Ammar N, Bailey JE, Davis RL, Shaban-Nejad A (2021). Using a personal health library-enabled mHealth recommender system for self-management of diabetes among underserved populations: use case for knowledge graphs and linked data. JMIR Form Res.

[30] Ammar N, Shaban-Nejad A (2020). Explainable artificial intelligence recommendation system by leveraging the semantics of adverse childhood experiences: proof-of-concept prototype development. JMIR Med Inform.

[31] Haynes RB, Sackett DL, Richardson WS, Rosenberg W, Langley RG (1997). Evidence-based Medicine: How to Practice and Teach EBM.

[32] Yeung CMA, Liccardi I, Lu K, Seneviratne O, Berners-Lee T (2009). Decentralization: the future of online social networking. W3C Workshop on Future of Social Networking Position Papers.

[33] Wang Z, Huang H, Cui L, Chen J, An J, Duan H, Ge H, Deng N (2020). Using natural language processing techniques to provide personalized educational materials for chronic disease patients in China: development and assessment of a knowledge-based health recommender system. JMIR Med Inform.

[34] Norris E, Finnerty AN, Hastings J, Stokes G, Michie S (2019). A scoping review of ontologies related to human behaviour change. Nat Hum Behav.

[35] Dragoni M, Bailoni T, Maimone R, Ecche C (2018). HeLiS: an ontology for supporting healthy lifestyles.

[36] Westerski A, Iglesias CA, Rico FT (2011). Linked opinions: describing sentiments on the structured web of data. https://oa.upm.es/13139/.

[37] Xu J, Kim S, Song M, Jeong M, Kim D, Kang J, Rousseau JF, Li X, Xu W, Torvik VI, Bu Y, Chen C, Ebeid IA, Li D, Ding Y (2020). Building a PubMed knowledge graph. Sci Data.

[38] Hassanzadeh O, Miller RJ (2015). Automatic curation of clinical trials data in LinkedCT.

[39] White BM, Melton C, Zareie P, Davis RL, Bednarczyk RA, Shaban-Nejad A (2023). Exploring celebrity influence on public attitude towards the COVID-19 pandemic: social media shared sentiment analysis. BMJ Health Care Inform.

